# 

**Published:** 1919-06

**Authors:** 


					﻿VOL. XXXIV.
June, 1919.
No. 12.
Texas
Medical
Journal
Entered at the postoffice at^^istia, Tesaevas secqn^-clasmnail matter
				

